# Suboptimal chemotherapy is an adverse prognostic factor in osteosarcoma

**DOI:** 10.1186/1477-7819-10-191

**Published:** 2012-09-17

**Authors:** Bicheng Yong, Pingxian Tan, Junqiang Yin, Changye Zou, Xianbiao Xie, Jin Wang, Gang Huang, Qianyong Wang, Jingnan Shen

**Affiliations:** 1 Musculoskeletal Oncology Department, First Affiliated Hospital of Sun Yat-Sen University, 14th floor #58 Zhongshan 2nd Road, Guangzhou, Guangdong, 510080, China

**Keywords:** Osteosarcoma, Suboptimal chemotherapy, Survival

## Abstract

**Background:**

We sought to determine whether suboptimal chemotherapy compromised the prognosis of osteosarcoma patients.

**Methods:**

A total of 132 eligible patients who underwent chemotherapy between 1998 and 2008 were identified in our database. Information regarding patient demographics, clinical characteristics, and survival status were extracted for analysis. Optimal chemotherapy was defined as receipt of ≥80% of the planned dose intensity of prescribed agents within the planned durations.

**Results:**

The use of optimal chemotherapy resulted in an overall survival benefit with *P =* 0.006. Patients who failed to complete the optimal chemotherapy protocol had a dismal prognosis of 30.8% overall survival over five years, whereas those who completed the optimal chemotherapy had an overall survival rate over five years of 65.3%. Based on multivariate analysis, patients who were treated with a suboptimal protocol had a higher risk of relapse, metastasis and mortality. The hazard ratio (HR) of recurrence or death for the suboptimal chemotherapy group was as high as 2.512 over that of the optimal chemotherapy group (HR = 2.512, 95% confidence interval = 1.242 to 3.729).

**Conclusions:**

Chemotherapy is a significant independent prognostic variable, and suboptimal chemotherapy was found to have a detrimental effect on the outcome of patients with osteosarcoma.

## Background

Osteosarcoma is the most common malignant bone tumor; it predominantly affects adolescents and continues to be an extremely aggressive disease despite the use of neoadjuvant chemotherapy protocols and wide-margin, limb-salvaging surgery [1]. The five-year overall survival (OS) rate of osteosarcoma patients of all ages has been reported to range from 53% to 58% in the United States and Europe [2].

An approximately 70% long-term event-free survival rate for osteosarcoma patients can currently be achieved by using the standard three-drug chemotherapy protocol that includes cisplatin, doxorubicin and high-dose methotrexate [3]. The addition of ifosfamide to a three-drug regimen has been extensively used and tested in several clinical trials [4-7]. In China, chemotherapy for osteosarcoma has lagged significantly behind other countries. Most chemotherapy treatments for osteosarcoma were adopted from other countries [8]. However, the phenomenon that patients often abandon treatment prematurely or choose to delay resuming chemotherapy is quite prevalent but is not seriously recognized in musculoskeletal oncology.

The present study aimed to evaluate the effect of incomplete or suboptimal chemotherapy on survival and to discuss possible reasons for lack of compliance to treatment. We found that suboptimal chemotherapy is an unfavorable prognostic factor in the overall survival (OS), event free survival (EFS), relapse free survival and metastasis free survival of osteosarcoma patients. Inadequate finances, poor patient-doctor relationship and psychological and behavioral compromise may contribute to suboptimal chemotherapy.

## Methods

### Patient eligibility

We retrospectively reviewed the medical charts of 410 osteosarcoma patients who were treated at our hospital between 1998 and 2008. The last follow-up for event-free survivors was in October, 2010. The minimum follow-up time was 24 months. The criteria for inclusion in the study were as follows: (1) Enneking stage IIB [9]; (2) no history of treatment except needle biopsy; (3) scheduled for neo- and adjuvant chemotherapy; (4) surgery at our institute; and (5) longer than 24 months of follow-up for event-free patients. Of the 410 patients, 367 were Enneking stage IIB. Patients were excluded for the following reasons: prior chemotherapy, surgery or open biopsy at another hospital (n = 32), no treatment (n = 23), chemotherapy alone (n = 63), or surgery alone (n = 9). In addition, patients who were followed for less than 24 months (n = 108) were excluded. Therefore, the final study population consisted of 132 patients; the clinicopathological characteristics of these patients are summarized in Table 1. Several variables were recorded, and the influence of the variables on survival, local recurrence, and metastasis were statistically evaluated. This study was approved by the Ethical Committee of First Affiliated Hospital, Sun Yat-Sen University.

**Table 1:** Demographic and clinical characteristics of the cohort

	**Number**	**Percent of total (100%)**
**Total patients**	**132**	**100**
Age		
≤11(F), ≤12(M)	24	18.2
12 to 14 (F), 13 to 15 (M)	34	25.8
15 to 39 (F), 16 to 39 (M)	70	53.0
≥40	4	3.0
Gender		
Male	93	70.5
Female	39	29.5
Location		
Distal femur	66	50.0
Proximal tibia	35	26.5
Proximal humerus	6	4.5
Proximal fibula	11	8.3
Proximal femur	4	3.0
Hip/pelvic	3	2.3
Others	7	5.3
Pathological fracture		
Yes	7	5.3
No	125	94.7
Subtype		
Osteoblast	103	78.0
Chondroblastic	13	9.8
Fibroblastic	6	4.5
Others	10	7.6
Chemotherapy		
Optimal chemotherapy	52	39.4
Suboptimal chemotherapy	80	60.6
Local recurrence		
Yes	27	20.5
No	105	79.5
Distal metastasis		
Yes	57	43.2
No	75	56.8

**F, female; M, Male**.

### Protocol description

**Figure 1:**
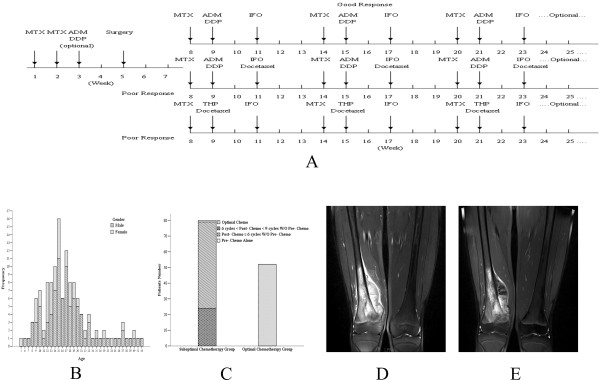
**A Optimal chemotherapy protocol.** MTX: methotrexate, DDP: cisplatin, ADM: adriamycin, IFO: ifosfamide. **B** Age and gender distribution in this cohort. **C** Proportion of patients receiving suboptimal chemotherapy and optimal chemotherapy. **D** Initial MRI of a patient with osteosarcoma of the right distal femur. T2-weighted MRI shows altered signal intensity involving the distal metaphysis, growth plate and medial condylar of the right femur with minimal necrosis. **E **Follow-up after two courses of MTX (8.5 g/m^2^) and one course of ADM (60 mg/m^2^) plus DDP (100 mg/m^2^) demonstrated a positive response to chemotherapy based on the extent of liquefactive necrosis and cystic changes of the soft tissue component despite the absence of a significant reduction in size. MRI magnetic resonance imaging.

Optimal chemotherapy was defined as follows: (1) the administration of methotrexate (MTX), cisplatin (DDP), adriamycin (ADM), ifosfamide (IFO) or their combinations; (2) the administration of MTX at a dose of 8 to 12 g/m^2^ (with leucovorin‘rescue’ commencing six hours after the initiation of the MTX infusion), cisplatin at a dose of 100 mg/m^2^ for four hours followed by 60 mg/m^2^ of adriamycin for 48 hours, and IFO at a dose of 2 to 3 g/m^2^ for five days with an injection of mesna (400 mg) after 12 hours of IFO administration; (3) one cycle of chemotherapy (MTX, DDP + ADM, IFO) that took place over 42 days; and (4) patients who received ≥80% of the planned dose intensity [10]. At least two courses of MTX were required for induction chemotherapy, and at least three cycles of triplets (MTX, DDP + ADM, IFO) were applied after definitive surgical therapy (Figure 1A). At our institute, IFO was introduced into our optimal protocol in 1999. Prior to that, postoperative chemotherapeutic regimens were the same as the preoperative regimens; after the inclusion of IFO, poor responders were administered IFO-containing regimens more frequently, while good responders were treated with the same regimen as for the preoperative therapy. The chemotherapy response was primarily determined based on physical evaluation and radiographic reassessment according to Response Evaluation Criteria in Solid Tumors (RECIST) criteria. A lesion in the same plane was consistently measured [11].

### Study definition

In the survival analysis, the primary end points were time until death, time until relapse, and time until metastasis. Overall survival (OS) was measured in days from the date of diagnosis to the date of death. Metastasis-free intervals were measured from the date of diagnosis to the date of metastasis (the metastatic location was either lung or bone) or death. The relapse-free interval was measured from the date of diagnosis to the date of relapse or death. Event-free survival (EFS) was measured from the date of diagnosis to the date of any significant event (metastasis, relapse or death). Patients who did not develop metastasis or relapse or remained alive at the final follow-up were censored at that time.

### Surgery

Surgical interventions for osteosarcoma included limb salvage surgery and amputation. Definitive surgical therapy required a wide or radical surgical margin. The options for postoperative reconstruction included modular prosthesis, extendible prosthesis, and autograft or allograft transplantation with plate or intramedullary interlocking nail. Amputation included simple amputation, rotationplasty amputation, and modified rotationplasty amputation. The surgical type was determined by the patient’s life expectancy, maturity of epiphysis, presence of pathological fracture, clinical and surgical classification, and the patient’s willingness and financial capacities.

### Follow-up

After completing chemotherapy, patients were followed up for two years with quarterly radiographs of the operated limb. Computed tomography (CT) of the chest was performed every three months for two years. Thereafter, the same protocol was repeated every six months for up to five years and then annually for up to the tenth year after surgery. All of the patients were followed up for at least two years after diagnosis unless they were deceased.

### Statistics

Both OS and EFS were determined based on the Kaplan-Meier Survival Analysis, and the results in the different groups were compared using the log-rank test. Only variables that were significantly different in the univariate study were entered into the multivariate analysis using the Cox proportional-hazards regression model. The analysis was performed using SPSS 13.0 and *P*-values of <0.05 were considered to be statistically significant.

## Results

### Demographic information

Among the total 410 patients, 132 met the inclusion criteria and their charts were reviewed for inclusion in the study group. The study population included 93 males and 39 females, with a median age of 16 years (range, 5 to 56 years); 34 (25.8%) patients had undergone the onset of their growth spurt (12 to 14 (females), 13 to 15 (males) (Figure 1B). The distal femur was the dominant location affected by osteosarcoma (50%), and the majority of the pathological sub-classifications were the osteoblast subtype (Table 1). Fifty-three (39.4%) cases underwent optimal chemotherapy while 80 (60.6%) cases did not complete the prescribed chemotherapy protocol. Among these, 2 cases underwent only preoperative chemotherapy, 23 cases underwent fewer than 9 cycles and more than 6 cycles of postoperative chemotherapy with or without preoperative chemotherapy, and 55 cases underwent fewer than 6 cycles of postoperative chemotherapy with or without preoperative chemotherapy (Figure 1C). Sixty-three (47.7%) events were recorded. Local recurrence occurred in 25 cases, including 3 cases of isolated local recurrence and 22 cases that eventually developed metastasis. Metastasis occurred in 57 cases, and the involved sites were the lung (55 cases), bone (1 case) and other sites (1 case).

### **Suboptimal chemotherapy was correlated with adverse overall survival, event free survival, relapse free survival and metastasis free surviv**al **in univariate analysis**

The five-year OS and EFS statistics are summarized in Table 2. The OS for the entire cohort was 43.5 ± 5.6%. However, patients who adhered to the optimal protocol had a five-year survival rate of 65.3 ± 7.3%, which was significantly better than the 30.8 ± 6.9% survival rate for the suboptimal chemotherapy group (*P =* 0.013). Similar results were obtained in EFS: 55.0 ± 9.0% patients were alive without disease in the optimal chemotherapy group, but only 31.1 ± 7.4% patients survived longer than five years without significant findings in the suboptimal chemotherapy group. Among the patients with metastases in the optimal chemotherapy group, 49.0 ± 7.2% survived for five years, while 30.4 ± 6.0% of those in the suboptimal chemotherapy group was still alive after five years of follow up. The outcome of patients with local recurrence in the optimal chemotherapy group was better than that of patients in the suboptimal chemotherapy group (five-year survival of 39.5 ± 6.7% versus 24.7 ± 6.1%) (Table 3).

**Table 2:** Overall survival and event-free survival

	**Overall survival**		**Event free survival**	
	**Five-year (%)**	***P***	**Five-year (%)**	***P***
Total patients	43.5 ± 5.6	N/A	40.3 ± 5.9	N/A
Age				
≤11(F), ≤12(M)	39.8 ± 14.2		34.8 ± 18.2	
12 to 14 (F), 13 to (M)	47.6 ± 10.3		43.9 ± 11.2	
15 to 39 (F), 16 to 39 (M)	42.2 ± 7.5		39.1 ± 7.8	
≥40	50.0 ± 35.4	0.396	37.5 ± 28.6	0.925
Gender				
Male	42.8 ± 6.4		37.8 ± 7.0	
Female	47.0 ± 10.8	0.104	46.2 ± 11.0	0.063
Location				
Distal femur	45.1 ± 8.1		38.6 ± 8.3	
Proximal tibia	45.9 ± 9.9		52.9 ± 10.7	
Proximal humerus	31.2 ± 45.7		66.7 ± 19.2	
Proximal fibula	25.0 ± 20.4		30.7 ± 17.1	
Proximal femur	50.0 ± 25.0		0	
hip/pelvic	0		0	
Other	57.1 ± 18.7	0.752	71.4 ± 17.1	0.696
Pathological fracture				
Yes	0		0	
No	46.7 ± 5.6	0.193	42.3 ± 5.9	0.759
Subtype				
Osteoblast	41.8 ± 5.9		39.2 ± 6.4	
Chondroblastic	35.2 ± 17.2		41.7 ± 19.9	
Fibroblastic	67.3 ± 27.0		66.7 ± 19.2	
Other	0	0.916	37.5 ± 28.6	0.723
Chemotherapy				
Optimal chemotherapy	65.3 ± 7.3		55.0 ± 9.0	
Suboptimal chemotherapy	30.8 ± 6.9	0.006*	31.1 ± 7.4	0.005*
Local recurrence				
Yes	23.2 ± 12.0		—	
No	49.3 ± 6.1	0.135	—	—
Distal metastasis				
Yes	18.2 ± 8.8	0.001*	—	
No	61.5 ± 6.5		—	—

*,*P*<0.05; F, female; M, male.

**Table 3:** Relapse free survival and metastasis free survival

	**Relapse free survival**		**Metastasis free survival**	
	**Five-year**	***P***	**Five-year**	***P***
Total patients	34.4 ± 4.0	N/A	33.6 ± 3.9	N/A
Age				
≤11(F), ≤12(M)	39.8 ± 14.2		26.6 ± 14.4	
12–14 (F), 13–15 (M)	37.3 ± 10.0		35.4 ± 9.6	
15–39 (F), 16–39 (M)	33.3 ± 7.0		33.2 ± 6.7	
≥40	37.5 ± 28.6	0.941	37.5 ± 28.6	0.952
Gender				
Male	32.1 ± 6.3		31.9 ± 5.9	
Female	41.9 ± 10.0	0.090	38.0 ± 9.7	0.053
Location				
Distal Femur	37.3 ± 7.7		32.4 ± 7.2	
Proximal Tibia	45.9 ± 9.9		41.8 ± 9.4	
Proximal humerus	25.0 ± 20.4		25.0 ± 20.4	
Proximal fibula	30.7 ± 17.1		46.0 ± 17.5	
Proximal Femur	25.0 ± 21.7		0	
Hip/Pelvic	0		0	
Others	0	0.454	57.1 ± 18.7	0.257
Pathological Fracture				
Yes	0		0	
No	37.1 ± 5.5	0.319	35.5 ± 5.2	0.386
Subtype				
Osteoblast	33.5 ± 5.7		31.9 ± 5.4	
Chondroblastic	29.3 ± 15.3		29.3 ± 15.3	
Fibroblastic	66.7 ± 19.2		83.3 ± 15.2	
Others	45.0 ± 32.2	0.257	33.8 ± 26.0	0.326
Chemotherapy				
Optimal Chemotherapy	49.0 ± 7.2		39.5 ± 6.7	
Suboptimal Chemotherapy	30.4 ± 6.0	0.009*	24.7 ± 6.1	0.005*
Local Recurrence				
Yes	—		13.7 ± 8.0	
No	—	—	40.3 ± 5.8	0.033*
Distal Metastasis				
Yes	21.1 ± 6.1		—	
No	50.1 ± 7.8	0.001*	—	—

*,*P*<0.05; F, female; M, male.

### Suboptimal chemotherapy is an independent unfavorable factor for overall survival, event free survival and metastasis free survival

**Figure 2:**
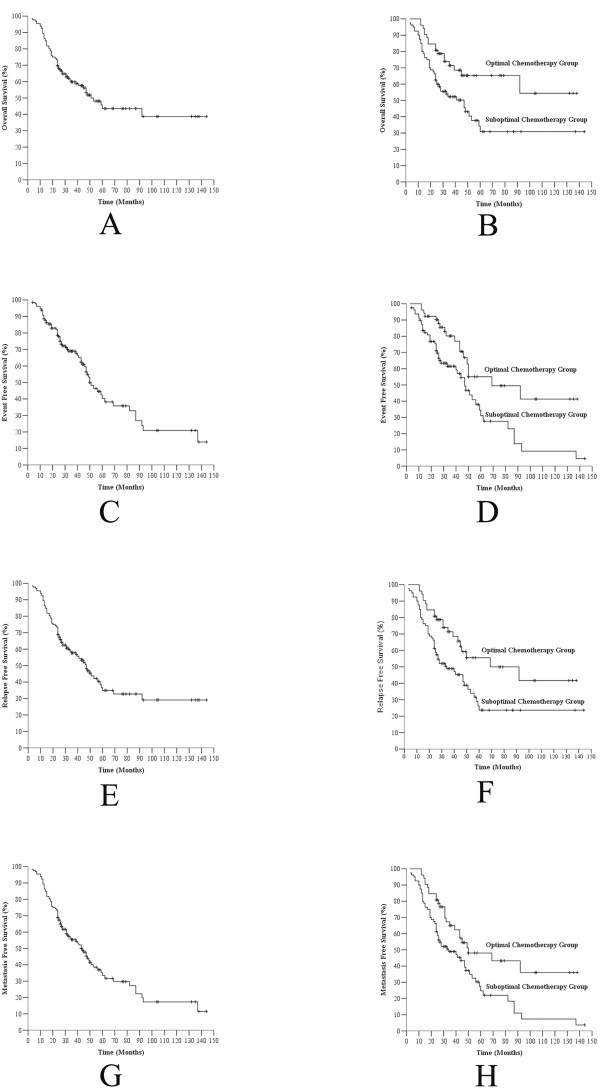
**A** Overall survival for all patients, compared to **B** Overall survival stratified by chemotherapy. **C** Event-free survival for all patients, compared to **D** Event-free survival stratified by chemotherapy. **E** Relapse-free survival for all patients, compared to **F** Relapse-free survival stratified by chemotherapy. **G** Metastasis-free survival for all patients, compared to **H** Metastasis-free survival stratified by chemotherapy.

Based on the multivariate analysis, chemotherapy was found to be an independent risk factor for OS, EFS and metastasis free survival. The risk of mortality and metastasis was approximately 1.8-fold higher in patients with suboptimal chemotherapy (hazard ratio (HR) = 1.804, 95% confidence interval (CI) = 1.016 to 3.204). Patients in the optimal chemotherapy group were more likely to experience recurrence (HR = 1.670, 95% CI = 0.987 to 2.825), although chemotherapy did not independently affect relapse free survival (*P =* 0.06) (Table 4). The Kaplan-Meier survival curves for the aforementioned data are shown in Figure 2.

**Table 4:** Cox proportional hazards model for the risk of death, relapse or metastasis, relapse alone and metastasis alone

	**Overall survival**			**Event free survival**			**Relapse free survival**			**Metastasis free survival**		
	**HR**	**95% CI**	***P***	**HR**	**95% CI**	***P***	**HR**	**95% CI**	***P*value**	**HR**	**95% CI**	***P*value**
Chemotherapy												
Suboptimal chemo	1.804	1.016 to 3.204		2.512	1.242 to 3.729		1.670	0.987 to 2.825		1.863	1.159 to 2.996	
Optimal chemo	1		0.04			0.006*	1		0.06	1		0.01
Local recurrence												
Yes	—	—	—	—	—	—	—	—	—	0.633	0.388 to 1.034	
No	—			—			—					0.07
Lung metastasis												
Yes	0.511	0.303-0.861	0.01*	—	—	—	0.509	0.312-0.829	0.007*	—	—	—
No	1			—			1			—		

*,*P*<0.05; CI,confidence interval; HR, hazard ratio.

## Discussion

The addition of neoadjuvant and adjuvant chemotherapy to radical surgery has been demonstrated to improve significantly the prognosis for osteosarcoma patients. Long-term survival in localized osteosarcoma has increased substantially from 10% to 20% (when surgery was performed as the only treatment) to 50% to 60% [12]. Five-year OS from 17 musculoskeletal oncology institutes in China ranges from 37.5% to 77.6% [13]. In the present study, the five-year OS for all patients was 43.5%, which is significantly lower than the international survival rate for these patients [14-16]. However, the five-year OS among patients who completed the optimal chemotherapy regimen was as high as 65.3% which is comparable to the international survival rate. There is a suggestion that many patients failed to complete the optimal chemotherapy protocol, and this phenomenon is also observed at other Chinese hospitals.

A combination drug protocol consisting of four drugs (DDP, ADM, MTX, and IFO) has been associated with an overall five-year survival rate of 44% to 65% [17,18]. International chemotherapy standards using these four drugs include the T12/T19 treatment developed by Rosen [19], the IOR-OS/N-2 treatment developed by Bacci and recommendations by the European Society for Medical Oncology (ESMO) in 2009 [20,21]. Although efforts have been made to identify optimized standard chemotherapy protocols that are suitable for the Chinese population, we do not yet have a nationwide sarcoma registry system that would support clinical trials with a large sample size to test standard protocols [8]. Our chemotherapy regimen was based on the four drugs described above and was a modified version of IOR-OS/N-2. The differences are as follows: (1) only two courses of MTX were required for induction chemotherapy and (2) IFO was routinely used after definitive surgery. For those who responded poorly to induction chemotherapy, we administered a more frequent IFO-containing regimen and/or added second-line drugs. The IOR-OS/N-2 protocol requires at least two cycles (four to six courses) for induction chemotherapy, but only two courses of preoperative medication are required in our protocol. We agree with Jeon and Bacci that the delayed removal of tumor increases the risk of systemic metastasis [22,23]. Furthermore, a smaller need exists for long-term neo-adjuvant therapy to bridge the gap between biopsy and resection due to the improvement of endoprostheses manufacturing. Third, the concern of non-compliance with long term induction chemotherapy must be considered. In our experience, two courses of MTX or two courses of MTX plus one course of ADM and DDP were adequate to achieve a good response to induction chemotherapy (Figure 1D and E). With this modified protocol, patients in the optimal chemotherapy group achieved an OS rate of 65.3% ± 7.3%, which is comparable to that in Bacci’s study [19].

In the univariate study, factors that were significantly different according to our stratifications are chemotherapy, local recurrence and lung metastasis.

Bajpai *et al*. showed that there was no significant difference in survival between the noncompliant versus compliant group in osteosarcoma [24] but in our study, patients who failed to complete the optimal chemotherapy protocol had less opportunity to achieve a stable disease status or complete remission. The reason why these two studies have different conclusions is obvious. Bajpai’s median follow up time is 7.9 months while our minimum follow-up time is 24 months. However, chemotherapy did not independently affect relapse-free survival (*P >*0.05). These data indicate that other factors (such as surgery) act in combination with chemotherapy to maintain local control of the disease. Picci has convincingly demonstrated that the local failure rate in osteosarcoma correlates with both the quality of the surgical margins and the extent of tumor response to induction therapy [25]. Surgical margins should always be seriously considered to be equally as important as chemotherapy.

Chemotherapy compliance was defined as adherence to a prescribed treatment regimen [26]. It can be affected by psychological and behavioral aspects as well as financial aspects. The former may include physician’s attitude, doctor-patient relationship, psychological support from others and religious convictions [27]. The latter was often unjustifiable and has social and economic roots. In China, the high price of drugs (including chemotherapy drugs and their rescue drugs) has long been blamed for medical services being unaffordable for less advantaged people and has triggered increasing complaints from the public [28]. Moreover, very expensive prosthesis (manufactured both domestically and abroad) causes some patients to discontinue chemotherapy after surgery. The current basic medical insurance system in China has not yet determined how to disseminate basic medical insurance coverage to benefit more patients with malignant tumors and how to reduce the proportion of personal health expenditures [29].

The possible solutions for unjustifiable chemotherapy non-compliance may include: (1) Increase the budget for medical services and restrain the price of chemotherapy drugs; (2) improve the national medical insurance system and enlarge the insurance coverage for malignant tumors; (3) encourage sponsorships and donations from society and set a charity fund for osteosarcoma patients; and (4) encourage domestic prosthesis development. The suggestions for justifiable non-compliance are as follow: (1) Strengthen the physician-patient relationship by providing adequate time for counseling, correct and accurate information interpretation, patient privacy protection and so on; (2) strengthen the family and social support system by providing personal and individualized care, chaplain services or psychological counseling; (3) strengthen patient education before and after hospitalization; and (4) strengthen the follow-up system by telephone, e-mail, and family visits to keep patients accountable for their timely therapy.

This study included some limitations. First, our study was limited by its retrospective nature; however, the issue under investigation could not be studied prospectively.

The prospective cohort study is of superior quality to a retrospective study in observational research; and the evidence level of a prospective study is higher than that of retrospective research. Nevertheless, it has the disadvantages of substantial expense, sensitivity to attrition and lengthy follow-up time. Desiring to improve our patients’ survival rates, we conducted this study to investigate the effects of suboptimal chemotherapy on osteosarcoma prognosis.

Second, our study was devised using data available from osteosarcoma patients at a single institution, and therefore, the total number of patients was relatively small. Our results would be more compelling if a larger number of patients being treated at different institutions had been included in the study, but our strict standards for inclusion naturally limited the size of our study. Our results also require validation in a large number of patients at different institutions. Finally, bias led by the inherent heterogeneity of the optimal chemotherapy regimen is difficult to eliminate.

## Conclusions

In summary, we investigated a cohort of osteosarcoma patients with long term follow-up, suboptimal chemotherapy compromised the outcome and prognosis of the subjects. Standardization of treatment is deemed necessary in the Chinese osteosarcoma population, and multicenter clinical trials to identify optimized chemotherapy drugs and protocols suitable for Chinese patients must be developed. Moreover, taking measures to improve patients’ compliance to treatment is of equal importance.

## Abbreviations

ADM: adriamycin; CI: confidence interval; DDP: cisplatin; EFS: eventfree survival; ESMO: European Society for Medical Oncology; HR: hazard ratio; IFO: ifosfamide; MTX: methotrexate; OS: overall survival.

## Competing interests

We declare that we have no conflicts of interest.

## Authors’ contributions

JNS and JW conceived the study. BCY, PXT, CYZ did the chart review. BCY and YQW followed up the patients and obtained the survival information. GH and BCY performed the statistical analysis and interpreted the results. BCY, XBX and JQY performed the literature review and wrote the manuscript. All authors read and approved the final manuscript.

## Authors’ information

Professor JNS is the director and chief surgeon of Musculoskeletal Department of First Affiliated Hospital of Sun Yat-Sen University, China.
